# Oxidative Glial Cell Damage Associated with White Matter Lesions in the Aging Human Brain

**DOI:** 10.1111/bpa.12216

**Published:** 2014-11-20

**Authors:** Sufana Al‐Mashhadi, Julie E. Simpson, Paul R. Heath, Mark Dickman, Gillian Forster, Fiona E. Matthews, Carol Brayne, Paul G. Ince, Stephen B. Wharton, Medical Research Council Cognitive Function and Ageing Study

**Affiliations:** ^1^ Sheffield Institute for Translational Neuroscience University of Sheffield Sheffield UK; ^2^ Department of Chemical and Biological Engineering University of Sheffield Sheffield UK; ^3^ Medical Research Council Biostatistics Unit University of Cambridge Cambridge UK; ^4^ Institute of Public Health University of Cambridge Cambridge UK; ^5^ King Fahad Medical City Riyadh Saudi Arabia

**Keywords:** dementia, DNA damage, ischemic white matter, white matter disease, white matter lesions

## Abstract

White matter lesions (WML) are common in brain aging and are associated with dementia. We aimed to investigate whether oxidative DNA damage and occur in WML and in apparently normal white matter in cases with lesions. Tissue from WML and control white matter from brains with lesions (controls lesional) and without lesions (controls non‐lesional) were obtained, using post‐mortem magnetic resonance imaging‐guided sampling, from the Medical Research Council Cognitive Function and Ageing Study. Oxidative damage was assessed by immunohistochemistry to 8‐hydroxy‐2′‐deoxoguanosine (8‐OHdG) and Western blotting for malondialdehyde. DNA response was assessed by phosphorylated histone H2AX (*γH2AX*), p53, senescence markers and by quantitative Reverse transcription polymerase chain reaction (RT‐PCR) panel for candidate DNA damage‐associated genes. 8‐OHdG was expressed in glia and endothelium, with increased expression in both WML and controls lesional compared with controls non‐lesional (*P* < 0.001). γH2Ax showed a similar, although attenuated difference among groups (*P* = 0.03). Expression of senescence‐associated β‐galactosidase and p16 suggested induction of senescence mechanisms in glia. Oxidative DNA damage and a DNA damage response are features of WML pathogenesis and suggest candidate mechanisms for glial dysfunction. Their expression in apparently normal white matter in cases with WML suggests that white matter dysfunction is not restricted to lesions. The role of this field‐effect lesion pathogenesis and cognitive impairment are areas to be defined.

## Introduction

White matter lesions (WML), which can be identified in life as areas of increased signal on T2‐weighted magnetic resonance imaging (MRI) scans, are a common feature of the aging brain 7, 23. They are associated with dementia, depression, impaired motor function and Alzheimer's disease, and may be risk factors for progression of mild cognitive impairment to dementia and to stroke 2, 3, 5, 8, 9, 17, 18, 30, 41, 43. The Medical Research Council Cognitive Function and Ageing Study (CFAS) is a longitudinal study of dementia and frailty in the population with a brain donation cohort, permitting assessment of neuropathologic correlates 26, 28, 45. In CFAS, WML have been found to be an independent predictor of cognitive impairment 12.

With a role in cognitive impairment, the pathogenesis of WML, and associated dysfunction in surrounding white matter are important questions. The association of vascular risk factors and vascular pathology with WML suggests that they are a manifestation of small vessel disease 25, 29, 31. Pathologically, WML consist of areas of white matter loss, or myelin attenuation, associated with astrogliosis, microgliosis and apoptosis of oligodendrocytes and astrocytes 1, 21, 34, 37. While they have been considered to represent infarction or incomplete infarction 10, 11, and there is histopathologic evidence for a role for hypoperfusion 14, their pathology and pathogenesis is likely to be more complex 45 and still remains poorly defined.

Studies of the pathology and pathogenesis of WML are hampered by the difficulty in recognizing these lesions in the autopsy brain. The CFAS approach has been to use post‐mortem MRI of brain slices to guide sampling of WML 13. This allows sampling of lesions, and of control white matter from cases with deep subcortical lesions (controls lesional, CL), which appears normal on routine histologic examination, and from cases without lesions (controls non‐lesional, CNL). These studies have defined the glial pathology of WML and shown up‐regulation of microglial activity 37, 39. Expression of hypoxia‐related molecules in WML supports a role for ischemia 14, but there is also evidence of blood–brain barrier leakage 38, suggesting that blood–brain barrier dysfunction may play a role in their pathogenesis. Periventricular WML appear to show some pathologic differences from WML in deep subcortical white matter; furthermore, lesions in these two locations may have different clinical effects 20. The current study focuses on deep subcortical WML (hereafter referred to simply as WML). Our previous work also demonstrated that apparently normal white matter from cases with lesions shows microglial activation and altered transcriptional profile, similar to those of the lesions 35, 37. This “field effect” suggests that WML (in a deep subcortical location) are associated with more widespread white matter abnormality.

Hypoxia/ischemia and inflammatory mechanisms, which have been demonstrated in this cohort, are associated with oxidative stress, which may impair cellular function. In this study we therefore hypothesized that oxidative DNA damage is a feature of WML pathology and pathogenesis that might impair glial cell function. We have therefore examined markers of oxidative damage and of a DNA damage response in WML and in the two control groups, with (CL) and without (CNL) lesions elsewhere in the white matter.

## Methods

### Cohort and tissues

Formalin‐fixed paraffin‐embedded (FFPE) and frozen samples of white matter were obtained from the MRC–CFAS, with Research Ethics Committee approval. Briefly, the study [previously reviewed in 45 ] is based around six UK centers, with recruitment from family practitioner registries based on age at entry (>65 years). For this study, FFPE samples were obtained from the Newcastle cohort and frozen samples from the Cambridge cohort. Using a similar case control structure to our previous white matter studies 37, 38, 39, the cohort consisted of: (i) samples of deep subcortical WML (DSCL); (ii) control white matter from cases with lesions elsewhere (CL); and (iii) controls from cases without WMLs (CNL). Tissue sampling from formalin‐fixed coronal cerebral slices to create FFPE blocks was guided by post‐mortem MRI scans to identify WML, as previously described 13. Immunohistochemistry was carried out on sections from 43 FFPE blocks derived from 40 individuals, 18 men, 22 women (WML 15 cases, mean age 86 range 74–89; CL 13 cases mean age 84.6 range 74–91; CNL 15 cases mean age 82.5 range 65–101). For frozen tissue samples, MRI scans of the formalin‐fixed contralateral brain hemispheres were used as a guide to map WML, as they tend to be distributed roughly symmetrically between the two brain hemispheres. Luxol fast blue stain for myelin loss and immunohistochemisty to the microglial marker CD68 were then performed on the frozen samples to confirm the lesion/non‐lesional status of the sampled blocks, as lesions show myelin loss and activated, amoeboid microglia. Twenty individuals (eight men, 12 women) provided 27 frozen blocks for analysis, comprising 11 WML (mean age 88.7 range 74–102), eight CNL (mean age 88 range 74–95) and eight CL (mean age 86.8 range 75–95).

### Immunohistochemistry

FFPE blocks were sectioned at 6 μm and frozen tissue sectioned at 8 μm before fixation in ice‐cold acetone at 4°C for 10 minutes. Immunohistochemistry was performed using a standard ABC method (Vector Laboratories, Peterborough, UK) and the signal visualized using 3,3′‐diaminobenzidine. A summary of the primary antibodies and their conditions, including antigen retrieval, is shown in Table 1. Negative controls consisted of sections incubated with isotype controls or with omission of the primary antibody.

**Table 1:** Antibodies: sources and conditions

Antibody	Species	Dilution and conditions	Antigen retrieval	Supplier
Primary antibodies for immunohistochemistry				
8‐OHdG	Mouse monoclonal	1/400 1 h RT	Pressure cooker access revelation (×10, pH 6.5)	Abcam, Cambridge UK
ɣH_2_AX	Rabbit monoclonal	1/1000 1 h RT	Pressure cooker EDTA pH8	R&D Systems, Abingdon UK
DNA‐PK	Mouse monoclonal	1/400 1 h RT	Pressure cooker EDTA pH8	Merck Biosciences Ltd, Nottingham UK
GFAP	Rabbit polyclonal	1/1000 1 h RT	Pressure cooker TSC pH6	DakoCytomation, Ely UK
OSP	Rabbit polyclonal	1/250 1 h RT	Pressure cooker TSC pH6	Abcam, Cambridge UK
CD68	Mouse monoclonal	1/100 1 h RT	10 min in microwave TSC pH6.3	Dako UK Ltd, Ely
Collagen IV	Mouse monoclonal	1/500 O/N at 4°C	10 min in microwave TSC pH6.3	Sigma‐Aldrich, Gillingham UK
P16	Mouse monoclonal	1/100 1 h RT	Pressure cooker EDTA pH8	Bio‐Genex, Fremont CA, USA
P53	Mouse monoclonal	1/50 O/N at 4°C	Pressure cooker EDTA pH8	Santa Cruz Biotechnology Inc., Heidelberg, Germany
P21	Mouse monoclonal	1/50 O/N at 4°C	Pressure cooker TSC pH6	Abcam, Cambridge UK
Primary antibodies for Western blotting				
ɣH_2_AX	Rabbit monoclonal	1/1000	NA	R&D systems, Abingdon UK
DNA‐PK	Mouse monoclonal	1/1000	NA	Abcam, Cambridge UK
MDA	Rabbit polyclonal	1/1000	NA	Cell Biolabs, Cambridge UK
β‐actin	Mouse monoclonal	1/1000	NA	Abcam, Cambridge UK
Secondary antibodies				
Goat anti‐mouse HRP	Mouse polyclonal	1/5000	NA	DakoCytomation, Ely UK
Goat anti‐rabbit HRP	Rabbit polyclonal	1/5000	NA	DakoCytomation, Ely UK

8‐OHdG = 8‐hydroxy‐2′‐deoxoguanosine; γH2Ax = Phosphorylated histone H2Ax; DNA‐PK = DNA protein kinase; EDTA = Ethylenediaminetetraacetic acid; GFAP = Glial Fibrillary Acidic Protein; HRP = horseradish peroxidise; NA = not applicable; OSP = oligodendrocytes; RT = room temperature; TSC = Tris‐saline‐citrate buffer.

Double‐staining experiments to localize 8‐hydroxy‐2′‐deoxoguanosine (8‐OHdG) to specific cell types were performed on FFPE sections. Staining for 8‐OHdG was performed using the same conditions as for single staining and the signal visualized using the diaminobenzidine chromogen. Sections were then placed in Tris‐buffered saline (TBS) buffer, incubated with 1.5% relevant normal sera for 1 h at room temperature (RT) before they were incubated with avidin‐biotin blocking kit (Vector Laboratories), according to the manufacturer's instructions. Sections were incubated with the second primary antibody for cell type [GFAP, CD68, oligodendrocyte‐specific protein (OSP), collagen IV] at 4°C overnight. Sections were washed thoroughly with TBS buffer and incubated with the relevant biotinylated secondary antibody for 1 h at RT, followed by streptavidin‐Tetramethylrhodamine isothiocyanate (TRITC) (1:100 in TBS) for an hour at (RT) in the dark. Sections were rinsed in TBS, air dried in the dark and mounted with Vectamount containing 4',6‐diamidino‐2‐phenylindole (DAPI) (Vector Laboratories). Sections were stored in the dark at 4°C and visualized in bright field to view oxidative damage (8‐OHdG) and a fluorescent field to co‐localize the damage with specific cellular phenotypes. Image capture was performed using Cell∧R (Olympus Biosystems, Watford, UK) and Leica DMI4000B, UK. Co‐localization of staining was analyzed using Corel Paint Shop Pro X (Corel, Maidenhead, UK).

### Quantification of 8‐OHdG and γH2Ax immunohistochemistry

**Figure 1:**
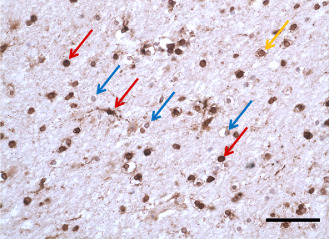
Immunohistochemistry to 8‐hydroxy‐2′‐deoxoguanosine showing strongly labeled nuclei (red arrows), unlabelled nuclei (blue arrows) and nuclei with faint reactivity (yellow arrow). Scale bar 50 μm.

Within each region (CNL, CL and WML), five random fields were captured using a Nikon Eclipse 80i microscope (Nikon UK, Kingston upon Thames). Images were transferred to a PowerPoint program where a grid was overlaid on each image (Figure 1A). The number of 8‐OHdG positive nuclei and total number of nuclei were assessed in the five fields for each case, allowing determination of percentage positive nuclei. For 8‐OHdG, only cells with positive nuclei, reflecting nuclear DNA oxidative damage, were assessed; cells with cytoplasmic only staining were not counted as positive.

### Histochemistry for senescence‐associated β‐galactosidase (SA‐β‐gal)

Prior to use, components of the SA‐β‐gal histochemical staining kit (Sigma‐Aldrich, Gillingham, UK) were thawed on ice and the X‐gal solution heated to 37°C for 1 h to activate. The frozen sections were warmed to RT for 5 minutes, fixed in ready‐made fixation solution for 6 minutes at RT followed by three rinses in 1× PBS. Freshly prepared staining mixture was then added, the sections were covered with parafilm and incubated overnight at 37°C. Sections were rinsed in TBS, counter‐stained with nuclear fast red for 30 s followed by a rinse in deionized water before they were dehydrated, cleared and mounted in DPX. SA‐β‐gal activity was microscopically detected by the presence of a blue, insoluble precipitate within the cell. SA‐β‐gal was assessed semi‐quantitatively in images captured at low magnification (×20 objective). The following scoring criteria were used: no positive cells (0); less than two positive cells per field (+); two to five positive cells (++); more than six isolated positive cells (+++).

To determine if SA‐β‐gal activity was associated with astrocytes, microglia and/or oligodendrocytes, GFAP‐, CD68‐ and OSP‐stained sections (using a standard ABC method as earlier) were double–labeled, respectively, with SA‐β‐gal. To determine the associations of SA‐β‐gal activity with cell cycle checkpoint protein p16, immunolabeled frozen sections (using ABC kit) were double‐labeled with SA‐β‐gal.

### Western blotting

Frozen white matter samples (approximately 0.5 g of tissue) from WML, CL and CNL were homogenized and sonicated for 30 s in buffer. Samples were then microfuged at 14 000 rpm for 30 minutes at 4°C. The supernatant was collected and stored at −80°C until required, while the pellet, which contained insoluble protein, was discarded. Bradford assay (reagents from Thermo Scientific, Loughborough, UK) was performed to determine protein concentration. Prior to blotting, protein samples were diluted in an appropriate volume of homogenate buffer to ensure equal protein concentrations (approximately 50 μg) for loading. Proteins were separated by sodium dodecylsulphate‐polyacrylamide gel electrophoresis [15% for γH2AX, 8% for MDA and 6% for DNA protein kinase (DNA‐PKcs) ] and transferred to a polyvinyl difluoride immobilin transfer membrane (Millipore, Dundee, UK). Membranes were incubated overnight with rabbit anti‐γH2AX (1:1000; R&D Systems, UK), rabbit anti‐MDA primary antibody (1:1000; Cell Biolabs, Cambridge, UK) or mouse anti‐DNA‐PKcs (1:500; Calbiochem, UK), followed by the appropriate horseradish peroxidise (HRP)‐linked secondary antibody (1:1000; DakoCytomation, Ely, UK). To confirm equal protein loading, the membrane was reprobed for β‐actin (1:5000; AbCam, Cambridge, UK). Proteins were detected using an enhanced chemiluminescence kit (Amersham, UK) for chemiluminescence based‐immunodetection of HRP. Membranes were developed and scanned using the G:box (Syngene, Cambridge, UK). Images were captured using the Intelli Chemi setting in the GeneSnap software (Syngene). Densitometric analysis was carried out in GeneTools (Syngene). Developed bands were manually framed in equally sized rectangular boxes that were manually designed to fit the largest band. Background was corrected by the software automatically. Raw data of the pixel intensity and the intensity of the bands in proportion to a defined control were calculated and the intensity of developed bands of interest normalized to the loading control.

### Reverse transcription quantitative polymerase chain reaction (RT‐qPCR) array

RNA extraction was performed from 50 μg of frozen tissue from each of 10 samples from the frozen cohort (three CNL, three CL, four WML). RNA was extracted using the Direct‐zol RNA MiniPrep kit (Zymo Research, Irvine, CA, USA). RNA quality was checked on the Agilent 2100 Bioanalyser (Agilent, Palo Alto, CA, USA) using an RNA 6000 Nano kit. RNA concentration was measured on the Nanodrop Spectrophotometer (ND1000, Labtech International, Uckfield, UK). RNA integrity number (RIN) numbers were 2.5, 3.1 and 4.2 for CNL cases, 2.9, 2.8 and 2.7 for CL and 2.4, 3, 3 and 1.1 for WML. 96 well RT‐qPCR arrays were obtained from Qiagen (Manchester, UK), custom designed for assessment of gene expression related to senescence and DNA damage (see Supplementary Figure S1). The selection of genes was made from those available on the commercial senescence and oxidative stress‐based array based on our prior knowledge and a literature search for likely candidates. RT‐qPCR was performed according to the manufacturer's instructions. Three housekeeping genes, comprising β‐actin, glyceraldehyde phosphate dehydrogenase and β‐2 microglobulin, were included in the arrays to enable normalization of the data. The average Ct and standard deviation (SD) for the three genes was determined for each group [average (SD) for: CNL 25.71 (1.54); CL 22.49 (0.47); WML 25.45 (1.56)] and the average used for normalization within each group. Further normalization to the CNL group (to a value of 1) was used to determine fold changes for each gene in the CL and WML groups. The plate was also designed with a genomic DNA control, which detects non‐transcribed genomic DNA contamination with a high level of sensitivity. A reverse‐transcription control tested the efficiency of the reverse‐transcription reaction and a positive PCR control (PPC) tested the polymerase chain reaction.

### Statistic analysis

Statistic analyses for the immunohistochemical data were carried out in Stata v12 (StataCorp LP, College Station TS, USA). Multiple readings by different readers were investigated using intra‐class correlation coefficient (ICC) for consistency. Quantitative comparisons among groups were compared using analysis of variance, taking into account non‐independent structure of the data and paired sampling of measures. *Post hoc* group differences were calculated using the Tukey–Kramer pairwise, after adjustment for multiple levels. All results were additionally checked using non‐parametric techniques and multilevel models. Associations were assessed using Spearman's correlation coefficient.

## Results

### Oxidative stress

**Figure 2:**
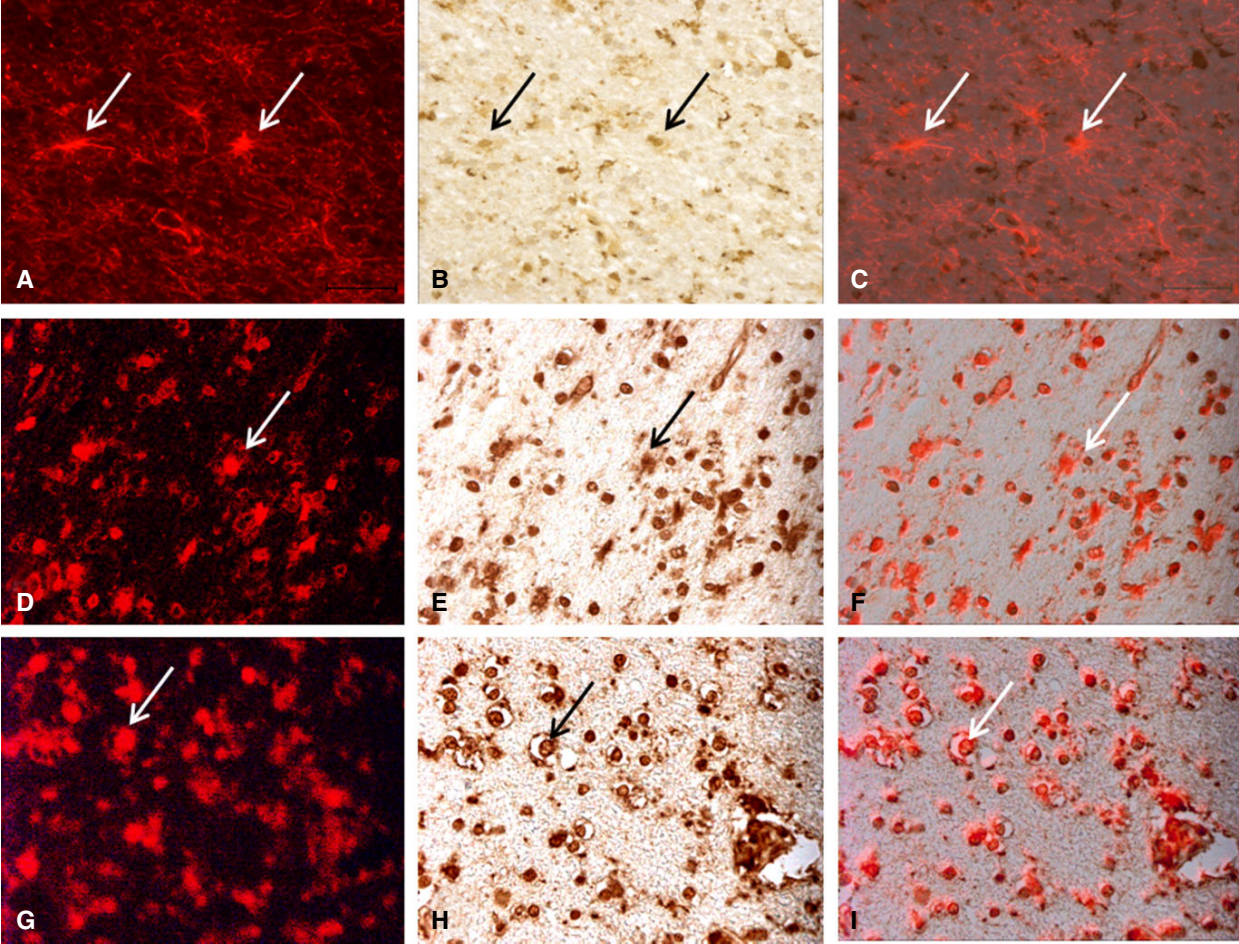
Double‐labeling studies for cell specific markers (red fluorescent label). **A.** GFAP; **D.** oligodendrocyte‐specific protein; **G.** CD68. 8‐hydroxy‐2′‐deoxoguanosine detected with 3,3'‐diaminobenzidine (DAB) label visualized under light microscopy (**B, E, H**). Merged images shown in **C**, **F** and **I**. Arrows identify example double‐labeled cells. Scale bar 50 μm.

Oxidative damage to nuclear DNA was assessed by immunohistochemistry to 8‐OHdG, with positivity ranging from faintly to strongly positive (Figure 1). In many cells, reactivity was also observed in the cytoplasm, presumably reflecting oxidative damage to RNA and/or mitochondrial DNA; cytoplasmic reactivity was not assessed further in this study. Double‐labeling immunohistochemistry demonstrated reactivity in astrocytes, oligodendrocyte, microglia and endothelial cells, although 8‐OHdG‐labelled astrocytes were uncommon (Figure 2).

**Figure 3:**
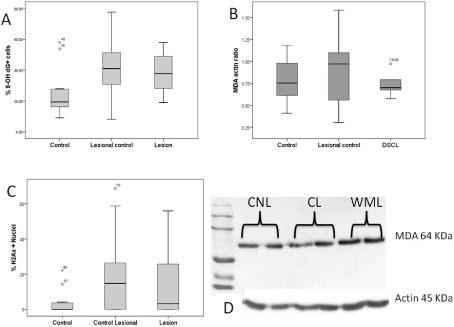
Box plots showing variation in 8‐hydroxy‐2′‐deoxoguanosine counts (**A**), malondialdehyde (MDA) protein by western blot (**B**) and γH2Ax counts among the three groups. **D.** Example Western blot for MDA showing expected size band at 64 kDa.

Variability in the intensity of nuclear staining inevitably resulted in some subjectivity in the assessment of positive cells. All cases were therefore quantified by two observers (SM and JS; Supporting Information Figure S2). There was a systematic difference between the level quantified by the two observers; however, they were consistent in scale (ICC = 0.75, 95% confidence interval 0.53–0.87, *P* < 0.001). Both measures were therefore used and adjusted for in the analysis, although the results are similar with the mean value. There was a significant difference in the percentage of 8‐OHdG‐positive nuclei amogn the three groups (Table 2, Figure 3A, *P* < 0.001), with differences seen between CNL and CL and between CL and WML (both *P* < 0.05), but not seen between CL and WML (*P* > 0.20).

**Table 2:** Quantification of markers

Marker		CNL	CL	WML
8‐OHdG % positive nuclei	Mean (SD)	24.7 (14.8)	41.8 (17.6)	38.5 (12.4)
	Median (IQR)	19.4 (12.1)	41.1 (24.3)	37.8 (22.1)
MDA	Mean (SD)	0.8 (0.28)	0.91 (0.44)	0.74 (0.15)
	Median (IQR)	0.75 (0.53)	0.97 (0.72)	0.70 (0.25)
γH2Ax % positive nuclei	Mean (SD)	3.6 (6.8)	20.6 (22.6)	14.1 (17.5)
	Median (IQR)	0 (3.9)	14.7 (31.5)	3.2 (26.0)
p53 % positive nuclei	Mean (SD)	15.4 (6.7)	20.9 (7.2)	12.2 (7.7)
	Median (IQR)	17.2 (14.7)	19.6 (13.9)	10.1 (12.7)

8‐OHdG = 8‐hydroxy‐2′‐deoxoguanosine; CNL = controls non‐lesional; IQR = interquartile range; MDA = malondialdehyde; SD = standard deviation; WML = white matter lesions.

Western blotting for malondialdehyde (MDA) revealed a band at the expected size of 64 kDa (Figure 3D). There was no statistic difference seen in the MDA levels among any of the groups (*P* = 0.65 (Table 2, Figure 3B).

### DNA damage response

**Figure 4:**
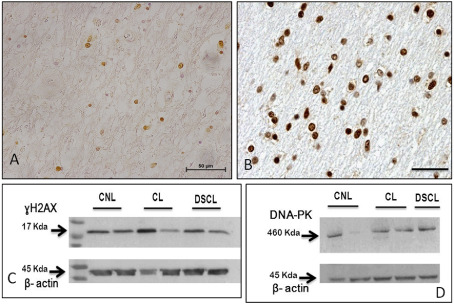
Immunohistochemistry showing nuclear expression of γH2Ax (**A**) and DNA protein kinase (**B**). Scale bars 50 μm. **C** and **D**. Western blots detecting bands of the expected sizes.

**Figure 5:**
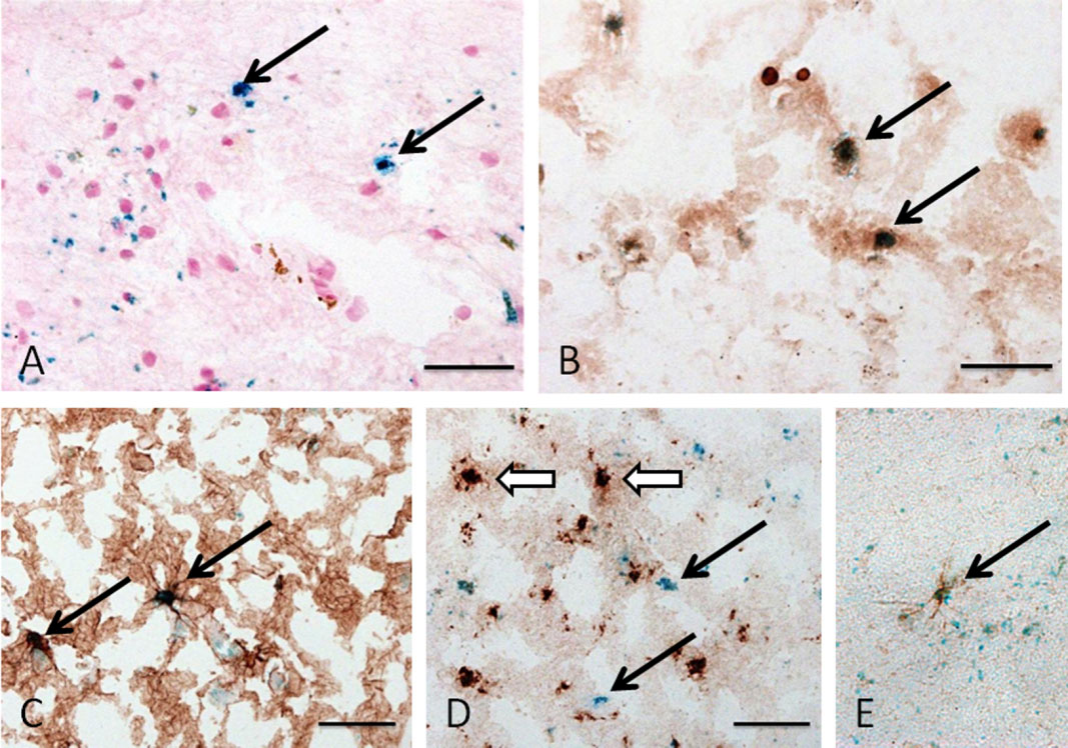
Expression of senescence markers. **A.** β‐gal expression in cells (arrows). **B.** β‐gal (blue) colocalized with oligodendrocyte‐specific protein (brown) indicating expression in oligodendrocytes (arrows). **C.** Colocalization of β‐gal with the astrocyte marker GFAP (arrows). **D.** β‐gal and CD68 double‐staining. β‐gal reactivity (arrows) does not colocalize with CD68 (open arrows). **E.** Colocalization of β‐gal with p16 (arrow).

The DNA damage response marker γH2Ax and the catalytic subunit of DNA‐PKcs showed widespread nuclear expression in white matter, with bands on Western blotting of 17 kDa and 480 kDa, respectively (Figure 4). The percentage of γH2Ax‐positive nuclei was assessed and quantified (Table 2). The γH2Ax and 8‐OHdG counts showed a moderate correlation (Spearman = 0.36). The γH2Ax counts showed a similar effect with lesion type to the 8‐OHdG, although the effects were slightly attenuated (*P* = 0.03) with CL being higher than CNL (*P* = 0.03), although the other *post hoc* adjusted comparisons were not significant (all *P* > 0.05). (Figure 3C). To determine whether nuclear oxidative DNA damage may have a detrimental effect on white matter cells, we examined the expression of markers for senescence. SA‐β‐gal activity was demonstrated histochemically in white matter, suggesting induction of senescence mechanisms (Figure 5A), but was not group‐specific. Double‐labeling studies demonstrated that SA‐β‐gal activity was present in astrocytes (GFAP+) and oligodendrocytes (OSP+), but was not observed in CD68‐positive microglia (Figure 5B–D).

**Figure 6:**
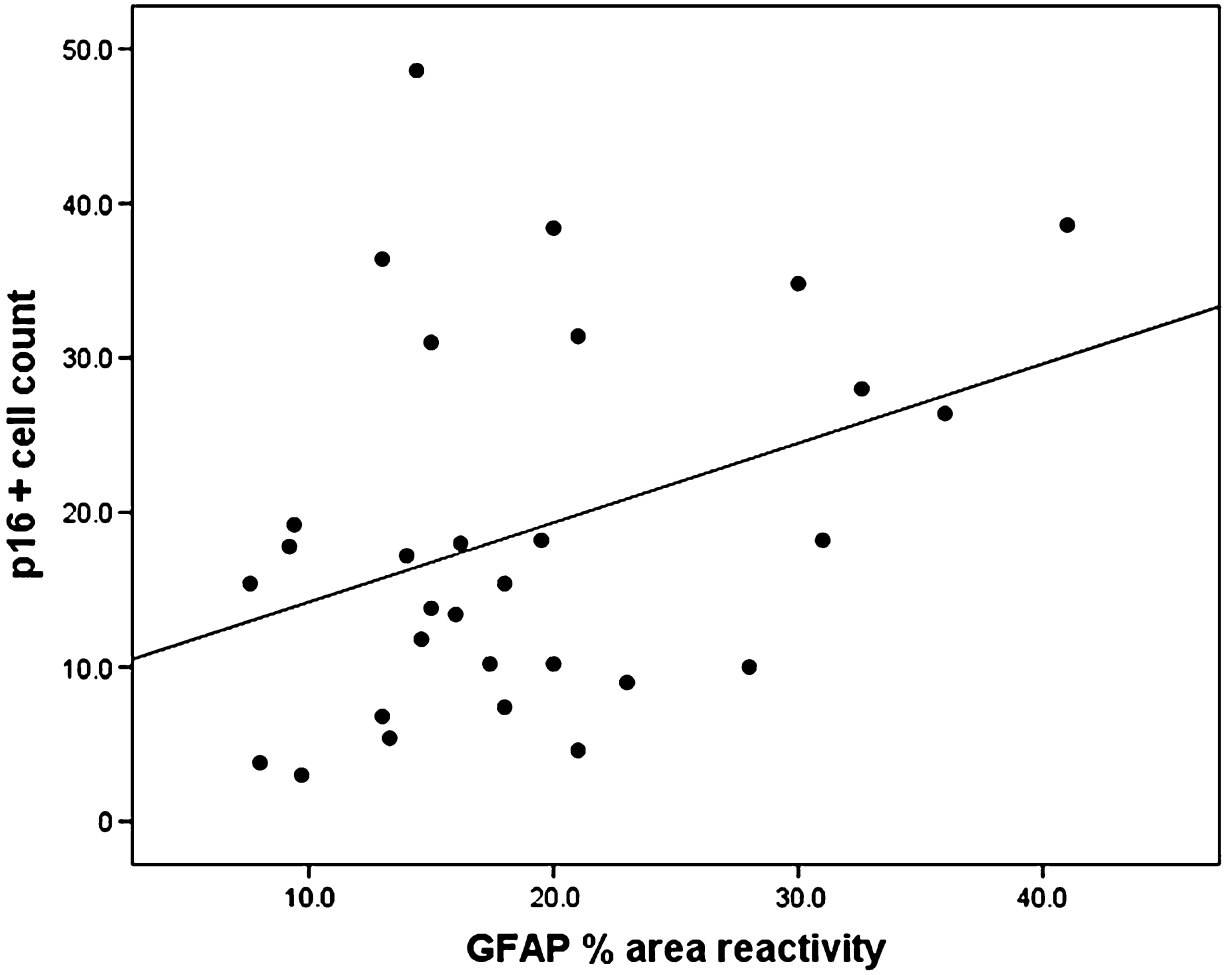
Scatterplot showing the association between p16 cell count and area immunoreactivity for GFAP.

To further assess the possibility of senescence induction, we examined expression of the cell cycle checkpoint protein p16. This was expressed in astroglial cells, where it co‐localized with SA‐β‐gal activity (Figure 5E). Quantification of SA‐β‐gal and p16 did not reveal differences between groups. Given the presence of p16 in the cytoplasm of astrocytes, we sought to determine whether p16 expression was associated with gliosis (irrespective of group). A weak relationship between p16 + cell count with GFAP area immunoreactivity was found (rho = 0.29, *P* = 0.11 Figure 6), but not in the p16 area immunoreactivity (rho = 0.17, *P* = 0.40).

**Figure 7:**
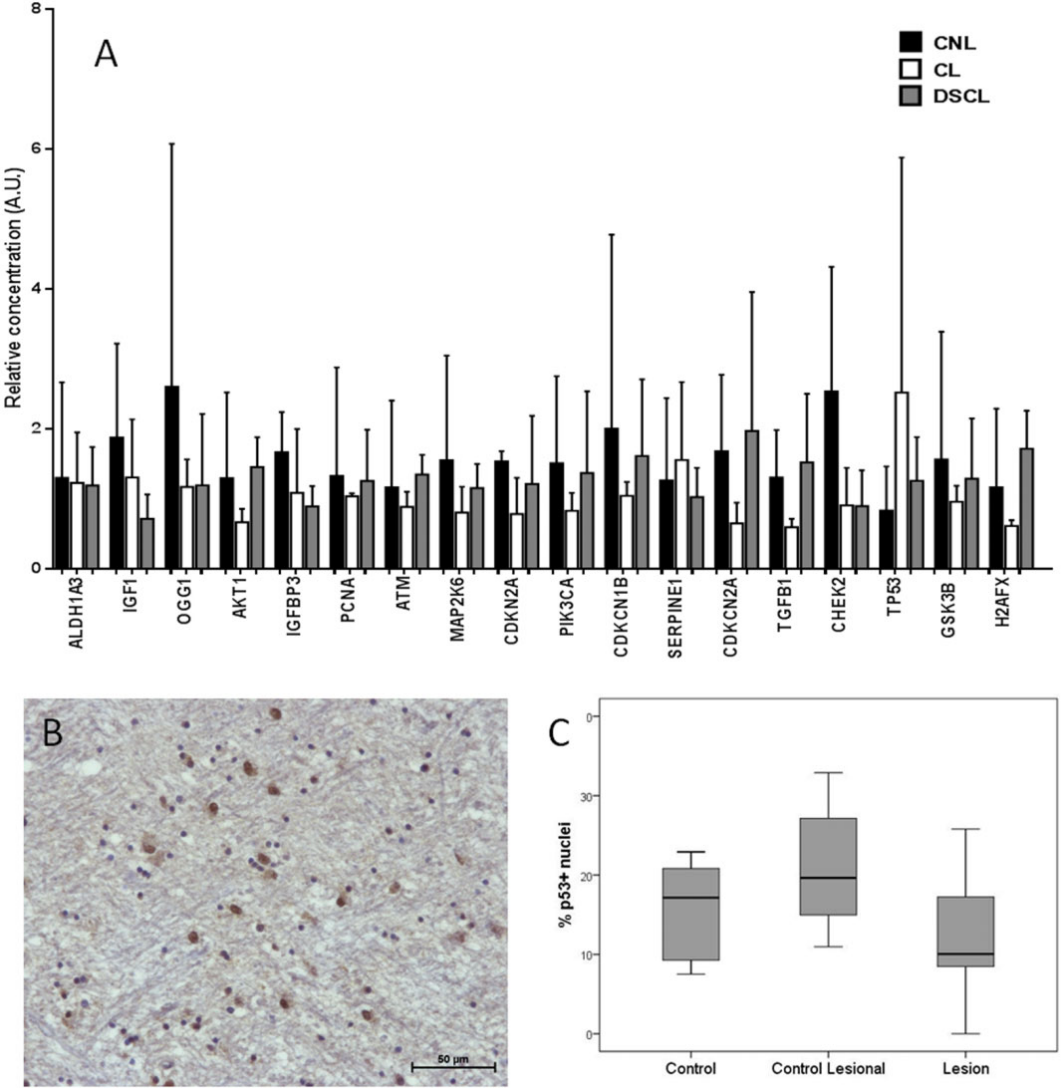
**A.** Variation in gene expression between control non‐lesional, control lesional (CL) and deep subcortical (here labeled DSCL) from RTqPCR array, Error bars represent standard deviations **B.** Expression of p53 (scale bar 50 μm). **C.** Boxplot showing variation in percentage of nuclei positive for p53 among the three groups, with higher values in the CL group.

We compared the expressions of several mRNA species using a customized qRT‐PCR panel (Supporting Information Figure S1) to seek further evidence for a pattern increased expression of genes related to DNA damage/senescence in CL and WML and to identify additional candidate senescence or DNA damage proteins (Figure 7A). For ease of comparison we compared changes in each gene in WML and CL, respectively, to CNL. In particular, expressions of *H2AX*, *TP53*, and *CDKN1B* (encodes p27) were increased by >1.5‐fold in WML compared with CNL, while expression of the *TP53* gene was also increased by >twofold in CL cases. Based on this RNA expression pattern, and the pivotal role of p53 in apoptosis and senescence, we selected p53 for further quantification. We therefore immunostained for p53 protein (Figure 7B) and quantified expression. Expression of p53 showed significant variation among groups (*P* = 0.01. Figure 7C). As for the RNA, the highest levels of p53 expression were in the CL group, and the expression pattern was similar to those of the other oxidative damage markers (see Figure 3). However, for p53 protein expression, levels were lowest in the lesions that showed lower counts than the CL group and the control group (*P* = 0.001, CL and *P* = 0.08 CNL).

## Discussion

The expression of markers of oxidative damage and of DNA damage response in WML and in CL suggests that oxidative stress plays a role in the pathogenesis of WML. The highest levels of expression were observed in apparently normal white matter from cases with lesions (CL), indicating that oxidative damage in white matter in cases with WML is extensive. Oxidative cell damage has previously been shown in white matter in multiple sclerosis, using immunohistochemistry to 8‐OHdG and lipid peroxidation markers, and associated with evidence of cellular injury 15. In that situation, injury correlated with inflammation. Our study now shows that similar cellular mechanisms may cause glial damage in the context of age‐related “ischemic” white matter pathology. Although not primarily inflammatory, as in multiple sclerosis, inflammation is a feature of WML and surrounding apparently normal white matter, where microglial activation is increased 37 and blood–brain barrier changes may have proinflammatory effects 32. So, inflammatory processes, in addition to effects of chronic ischemia and aging effects are possible contributors to oxidative DNA damage in ischemic white matter pathology.

The principal marker selected for this study was 8‐OHdG, an oxidative modification to a base that has been widely used as a marker of oxidative DNA damage, including in studies of aging and Alzheimer's disease, because of its relative sensitivity resulting from its low oxidation potential 27, 40. Antibodies to this marker also label oxidative damage to RNA and mitochondrial DNA, but in this study, as we were interested in the nuclear DNA damage response, we have focused on nuclear expression. A limitation of our quantification is that we counted immunopositive cells, but once a detection threshold is reached, this does not take account of the extent of DNA base modification within a particular cell, and so may underestimate the real variability. The pattern of highest levels of damage in lesional control tissue was also supported by assessment of MDA, a marker of lipid peroxidation, and γH2Ax, a histone that is phosphorylated in response to DNA double strand breaks, markers that we have previously used in studies in CFAS 36. Expression of MDA and γH2Ax did not reach significance, presumably related to the extent of intrinsic inter‐case variation in human autopsy material and limited sample size. However, they showed the same pattern of variation among groups as the 8‐OHdG, while the good correlation between 8‐OHdG and γH2Ax is consistent with the induction of a DNA damage response in cells with the oxidative nucleic acid modification.

Higher levels of oxidative/DNA damage were seen in lesional controls than in the WML themselves. This may suggest that pathogenic mechanisms are more active in surrounding apparently normal white matter than in the established lesions. We have previously shown microglial activation in lesional control white matter 37, while gene microarray studies show similar pathway alterations in lesional control white matter and WML 35. We therefore suggested a “field‐effect,” whereby WML are association with more widespread white matter dysfunction. The present results suggest that oxidative stress and DNA damage may contribute to this environment, causing glial cell dysfunction in this non‐lesional white matter. The relationship of this to lesion pathogenesis, particularly whether these changes provide an environment in which MRI‐recognizable lesions develop or whether it is secondary to the lesions, and its contribution to cognitive dysfunction remain to be determined.

Double‐labeling studies demonstrated DNA damage in glia and endothelial cells. Expression in individual cell types is an important question, but our double‐labeling was not sufficiently clear to allow reliable quantitation in these preparation. We therefore did not attempt to quantify the cell types separately. 8‐OHdG expression was uncommon in GFAP‐positive astrocytes, paralleling findings in multiple sclerosis where most 8‐OHdG cells were oligodendrocytes 15.

DNA damage can cause cell dysfunction or loss via apoptosis, and a persistent DNA damage response can induce senescence 44. The expression array study further supported increased expression of genes associated with DNA damage, particularly p53, which can mediate apoptosis, cell cycle processes and senescence following DNA damage 16.

SA‐β‐gal activity has been considered to be a marker of senescence in tissues. Senescence as a process was defined initially as a cell culture phenotype involving cessation of replication (replicative senescence), but it is becoming recognized in human tissue and senescent cells have recently been identified in neurons in brain 19, 42 and in cortical astrocytes 36. The expression of SA‐β‐gal now identified in white matter astrocytes and oligodendrocytes thus suggests induction of senescence mechanisms in glial cells. This was supported in astrocytes by the expression of the cell cycle checkpoint protein p16, which is involved in some senescence induction pathways 42, and which appeared to co‐localize with SA‐β‐gal in these cells. Astrocytes have been suggested to show features of senescence and a senescence‐associated secretory phenotype in association with GFAP up‐regulation in brain aging 33. The co‐expression of p16 and SA‐β‐gal in our study, and the suggestion of an association between p16 and gliosis, raises the question of whether p16 is involved in induction of gliosis and senescence in these cells. It should be noted, although, that cells that double‐stained for 8‐OHdG and GFAP were uncommon, so it is possible that induction of senescence in astrocytes in white matter may involve mechanisms other than 8‐OHdG‐associated DNA damage.

Quantification of SA‐β‐gal expression was difficult in these tissues, as has been previously noted in tissue sections 24, and we did not demonstrate clear differences among our groups. Therefore, at this stage, we cannot be certain of its relation to lesions, as compared with other potential drivers, such as age. However, senescent cells are not only dysfunctional, but may also contribute to tissue damage through the senescence‐associated secretory phenotype, which can have various effects within the tissue including pro‐inflammatory effects 6, 22. It therefore has potentially important implications for age‐related WMLs through a variety of possible mechanisms, including loss of glial support for myelin and axons, and for the induction of pro‐inflammatory environment and microglia.

Human autopsy‐based studies necessarily have a number of limitations, related to inherent inter‐individual variability and peri/post‐mortem factors. In addition the cohort size was relatively small. This may have masked differences between the groups for some of the markers. While a strength of the CFAS cohort is its population base, it should also be noted that the cases here were pre‐selected to represent WML, CL and CNL, so this study is not population representative, but is in essence a case control study nested within the CFAS population neuropathology cohort. We also do not yet have detailed data on axonal pathology in white matter, so that we are not able in this study to take account of interactions with axonal damage that might be arising secondary to cortical pathology, such as Alzheimer's, which can produce white matter damage 4.

In conclusion, oxidative DNA damage and a DNA damage response are features of the pathogenesis of WML and more so of the apparently normal white matter in cases with lesions, contributing to the concept that WML are associated with a field‐effect of white matter abnormality. Activation of downstream processes, such as senescence, are candidate mechanisms for altered glial function in white matter. Age‐related WML pathogenesis therefore involves active cellular processes, especially in surrounding white matter, which need to be understood as they may be molecular targets for improving white matter function and repair, and for preventing new lesions.

## Supporting information


**Figure S1.** Genes included in the customized RT‐qPCR array.
**Figure S2.** Counting of 8‐OHdG‐positive cells. **A.** Grid overlay to facilitate counting. **B.** Bland–Altmann plot showing variation in counts between two observers; the red line represents the mean difference in counts between observers while blue lines are the limits of agreement (mean difference ± 2 SD of mean difference). **C.** Correlation between two observers showing very good agreement for ranking of cases according to 8‐OHdG counts.
