# Supplementary figures and images for: Oxidative Glial Cell Damage Associated with White Matter Lesions in the Aging Human Brain
Source: Brain Pathol. 2014 Nov 20;25(5):565–74. doi: 10.1111/bpa.12216 (PMC4861214; doi:10.1111/bpa.12216)

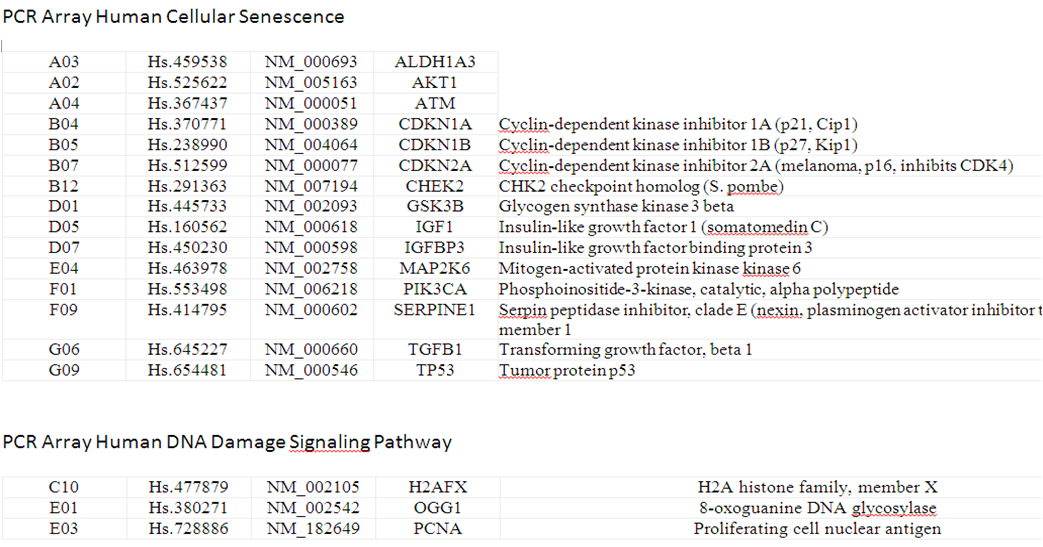

Supplement: Supplementary file 1 — Figure S1. Genes included in the customized RT‐qPCR array. Figure S2. Counting of 8‐OHdG‐positive cells. A. Grid overlay to facilitate counting. B. Bland–Altmann plot showing variation in counts between two observers; the red line represents the mean difference in counts between observers while blue lines are the limits of agreement (mean difference ± 2 SD of mean difference). C. Correlation between two observers showing very good agreement for ranking of cases according to 8‐OHdG counts. [file BPA-25-565-s001.zip › BPA_12216-supp-0001-Suppl Fig 1.tif]

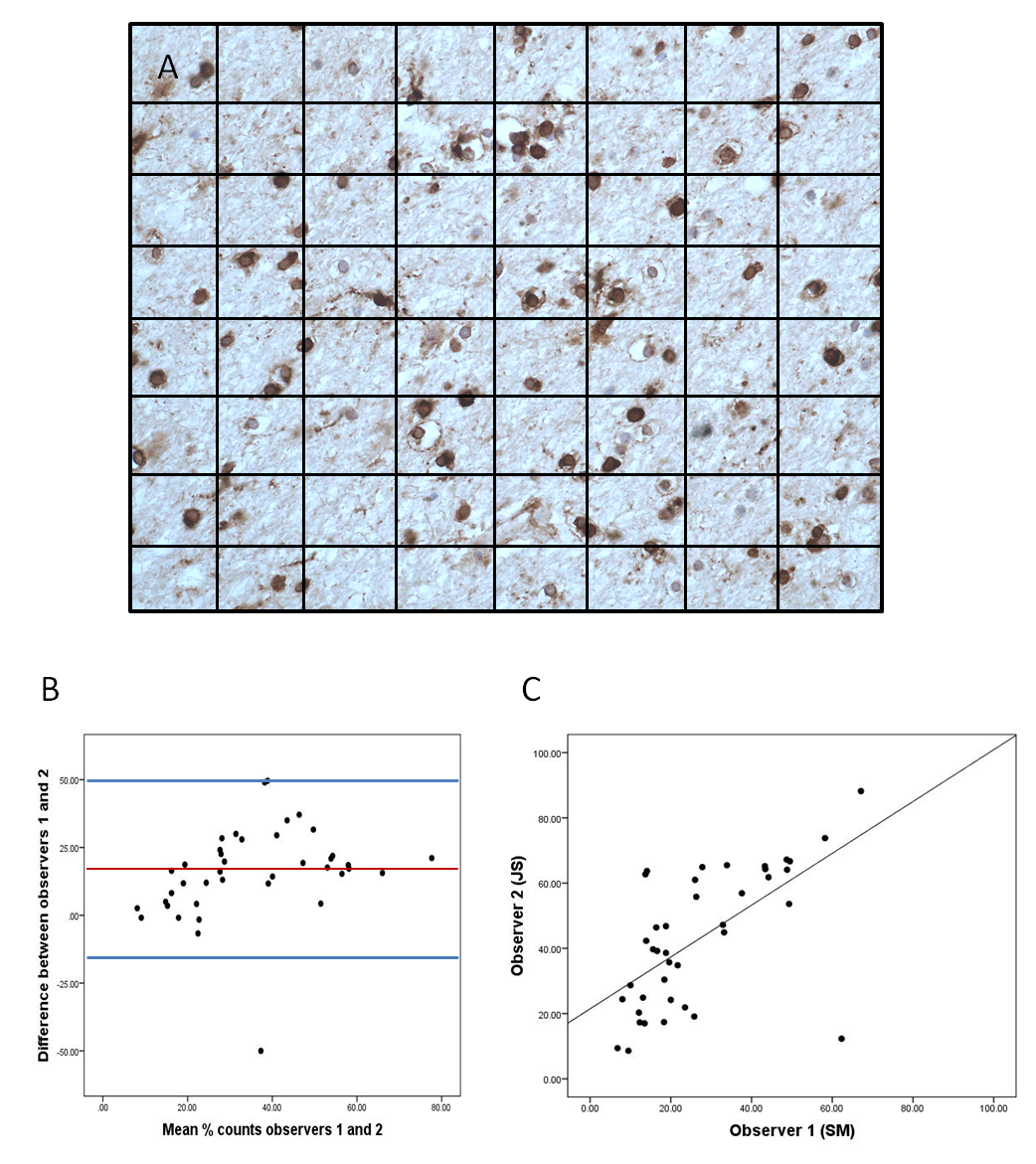

Supplement: Supplementary file 1 — Figure S1. Genes included in the customized RT‐qPCR array. Figure S2. Counting of 8‐OHdG‐positive cells. A. Grid overlay to facilitate counting. B. Bland–Altmann plot showing variation in counts between two observers; the red line represents the mean difference in counts between observers while blue lines are the limits of agreement (mean difference ± 2 SD of mean difference). C. Correlation between two observers showing very good agreement for ranking of cases according to 8‐OHdG counts. [file BPA-25-565-s001.zip › BPA_12216-supp-0002-Suppl Fig 2.tif]
